# Spinal Myxopapillary Ependymoma Presenting With Isolated Intracranial Hypertension: A Case Report

**DOI:** 10.7759/cureus.87616

**Published:** 2025-07-09

**Authors:** Emmanuel T Oke, Ekene Nnamani, Suhaib Abualsaud, Uchenna Nnamani

**Affiliations:** 1 Neurosurgery, The James Cook University Hospital, Middlesbrough, GBR; 2 General Practice, General Hospital, Umunze, NGA

**Keywords:** conus medullaris tumor, intradural extramedullary lumbar spinal cord tumor, myxopapillary ependymoma, papilledema, raised intracranial pressure

## Abstract

Myxopapillary ependymomas (MPEs) are rare, slow-growing spinal tumors that arise from the filum terminale and are classified as World Health Organization (WHO) Grade 2 tumors. They typically present with features of lower spinal cord or cauda equina compression, such as radiculopathy, limb weakness, or sphincter dysfunction. Presentation with features of increased intracranial pressure (IIP) is rare and has been reported in a few isolated cases. We present a case of a 21-year-old woman who presented with a two-week history of worsening headaches, neck stiffness, nausea, double vision, and tinnitus. Physical examination revealed bilateral abducens nerve palsies and papilledema, and lumbar puncture confirmed elevated cerebrospinal fluid (CSF) opening pressure. Brain imaging revealed features of IIP and showed superficial siderosis. In contrast, the spinal MRI scan revealed an intradural, extramedullary tumor at L1-L2 arising from the filum terminale, consistent with MPE. Surgical excision via L1-L3 laminectomy led to symptom resolution, and tumor histology confirmed a WHO Grade 2 MPE. The patient made a full recovery with no residual tumor on the three-month postoperative MRI. This case highlights a rare presentation of spinal MPE manifesting as IIP without typical lower limb neurology. The pathophysiology may involve elevated CSF protein and chronic subarachnoid haemorrhage, impairing CSF absorption. Spinal imaging should be considered in cases of unexplained intracranial hypertension, particularly when brain imaging is inconclusive.

## Introduction

Spinal cord myxopapillary ependymomas (MPEs) are slow-growing tumors from the filum terminale in the conus medullaris and cauda equina [1]. They are benign and classified as Grade 2 tumors, according to the World Health Organization (WHO) Classification of Central Nervous System tumors [2,3]. They make up 13% of spinal ependymomas and account for 90% of all tumors in the conus medullaris [3]. MPE has an incidence of about 0.05-0.08 per 100,000 persons each year, and it is predominant in males. Also, 10%-20% of this disease has been seen in the pediatric population [4]. They are often seen in adults with a modal age of 30-50 years and can present with radicular back and extremity pain, bladder and bowel dysfunction, as well as lower extremity weakness, sensory loss, and paresthesia [3,5]. 

We present a case of a 21-year-old lady who had features suggestive of increased intracranial pressure (IIP), and spinal imaging revealed an intradural tumor arising from the filum terminalis.

## Case presentation

A 21-year-old lady presented with a two-week history of worsening headaches, nausea, neck stiffness, tinnitus, and double vision. There was no fever or seizures, radicular back pain, limb weakness, sensory abnormalities, bladder, or bowel dysfunction. 

Neurological examination revealed bilateral abducens nerve palsies, intact limb neurology, and bilateral papilledema.

**Figure 1:**
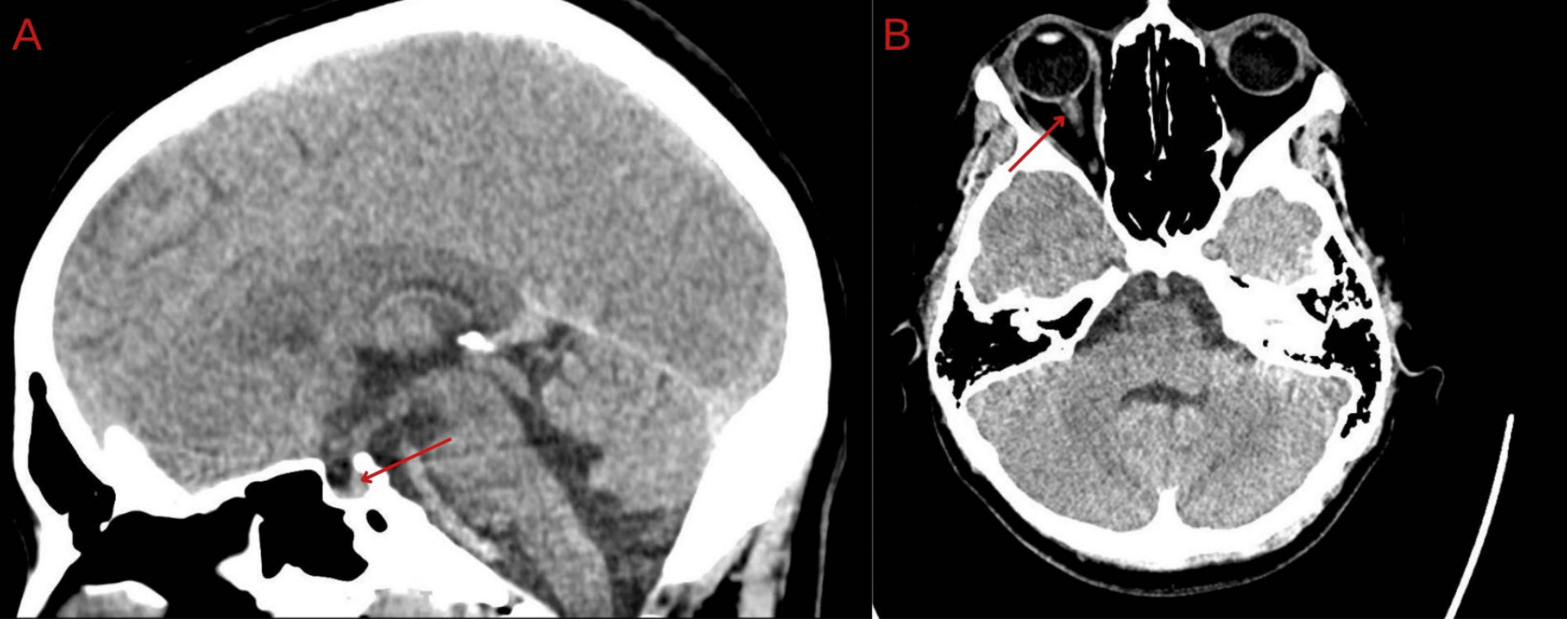
Non-contrast brain CT scan: sagittal view (A) shows a flattened pituitary gland, and axial view (B) demonstrates mild widening of the optic nerve sheaths.

A non-contrast brain CT scan was suggestive of intracranial hypertension with findings of supratentorial effacement, mild widening of the optic nerve sheaths, flattened pituitary gland, and small transverse sinuses, while the CT venogram showed no abnormalities (Figure 1).

Lumbar puncture revealed CSF opening pressure was 30.5 cmH2O. 

**Figure 2:**
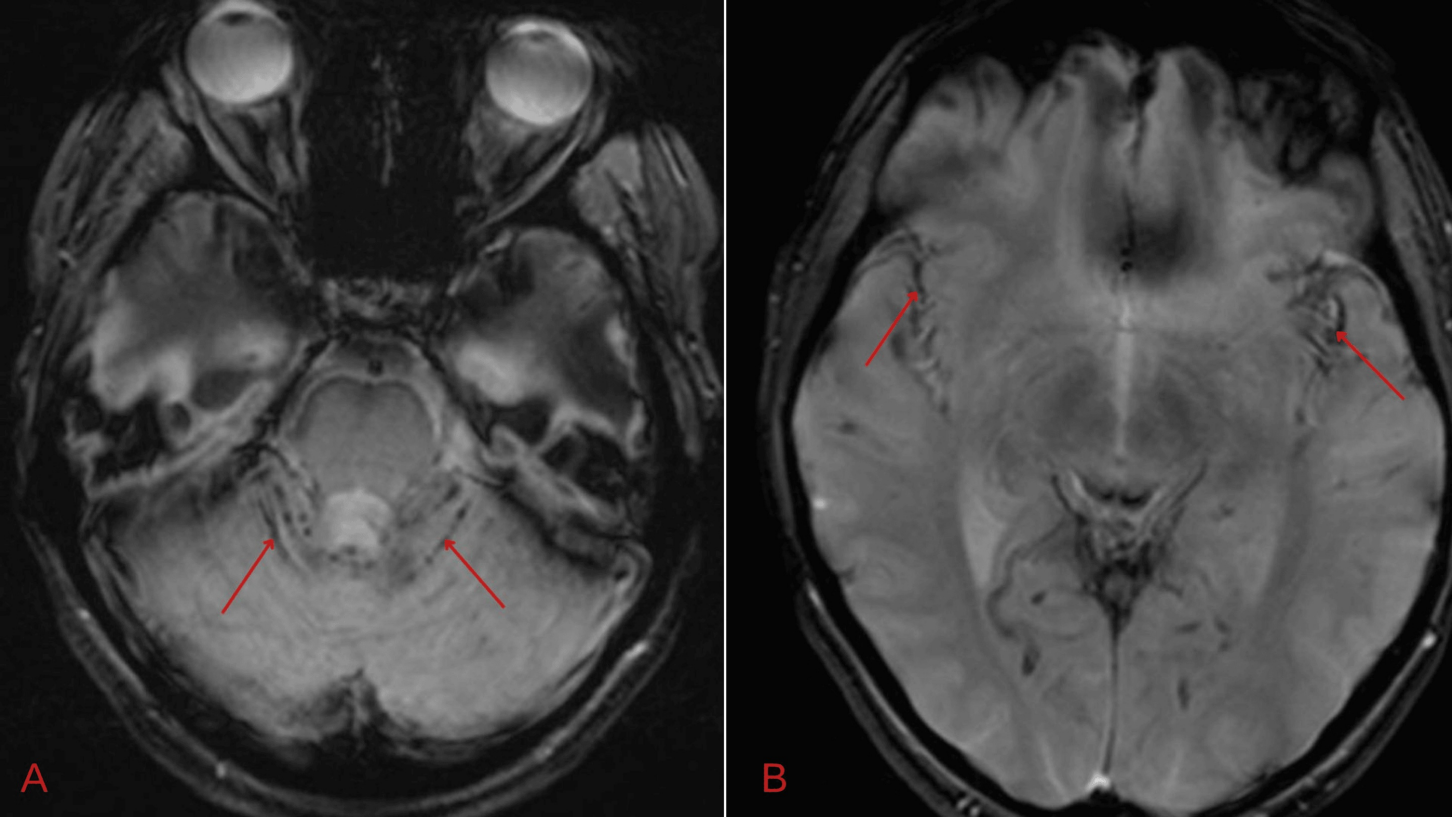
MRI brain axial Gradient Echo T2 sequence revealing superficial siderosis in the posterior fossa (A) and Sylvian fissures (B). MRI, magnetic resonance imaging

Brain magnetic resonance imaging (MRI) and magnetic resonance angiography (MRA) revealed superficial siderosis in the posterior fossa, as well as in the interhemispheric and Sylvian fissures, likely indicating repeated subarachnoid hemorrhages (Figure 2). 

**Figure 3:**
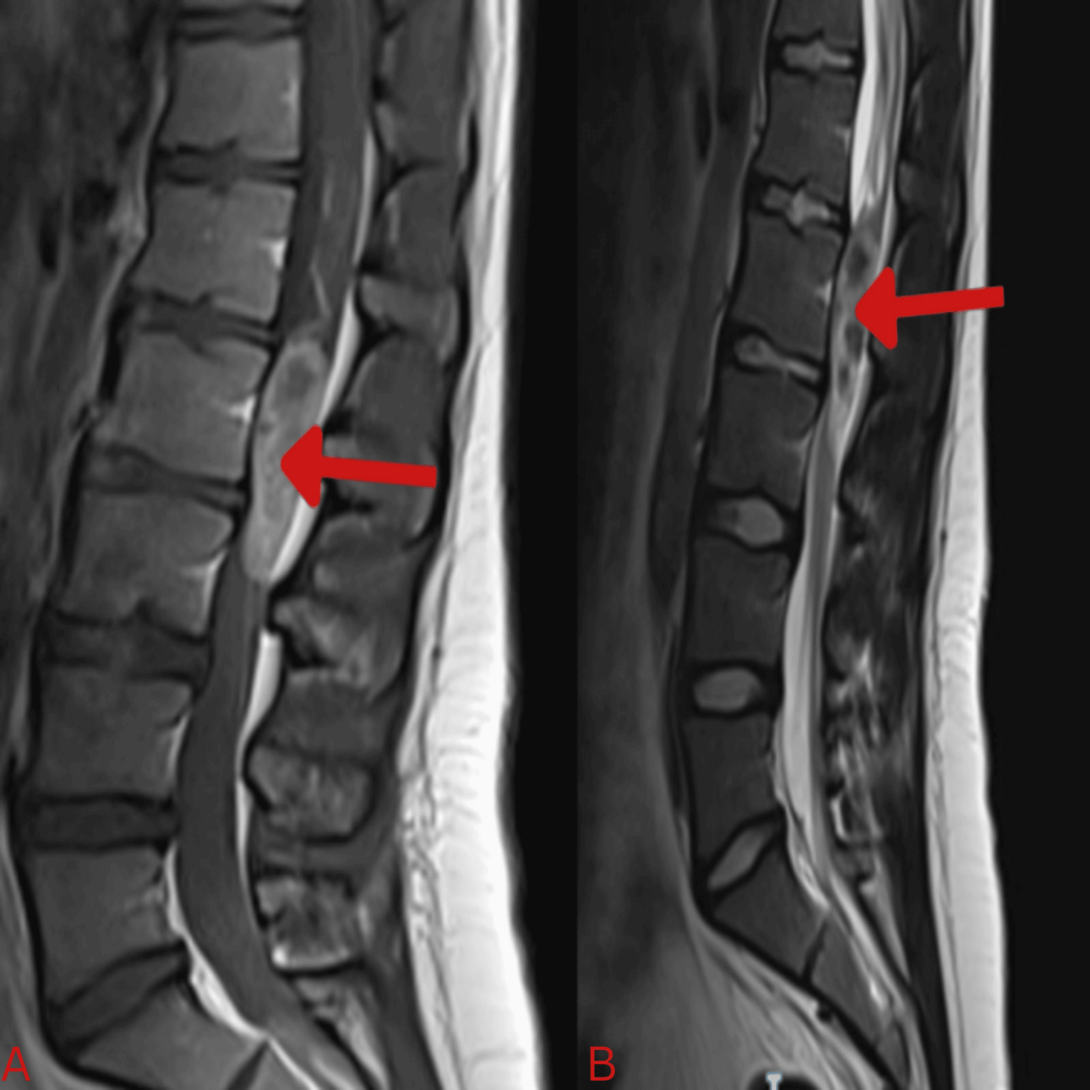
Sagittal T1(A) and T2(B) with contrast MRI of lumbo-sacral spine revealing the lesion, its radiological characteristics and extent. MRI, magnetic resonance imaging

A spinal MRI scan showed an intensely enhancing intradural extramedullary lesion extending from the L1 to L2 intervertebral disc levels. It originated from the filum terminale and completely occupied the spinal canal, displacing the cauda equina nerve roots. It showed low signal intensity on T1-weighted images (T1WI) and heterogeneous, predominantly high signal intensity on T2-weighted and STIR images, with central areas of low intensity suggestive of internal cysts containing proteinaceous material. A fluid-fluid level was seen in the distal thecal sac, with the dependent fluid showing a signal intensity pattern consistent with chronic blood degradation products (Figure 3). 

Management

The findings from the image was suggestive of MPE arising from the filium terminale. A decision was made to surgically excise the lesion. The patient underwent an L1-L3 laminectomy and removal of the lumbar intradural extramedullary tumor under intraoperative neurophysiological monitoring, with postoperative resolution of symptoms. 

Histopathology

**Figure 4:**
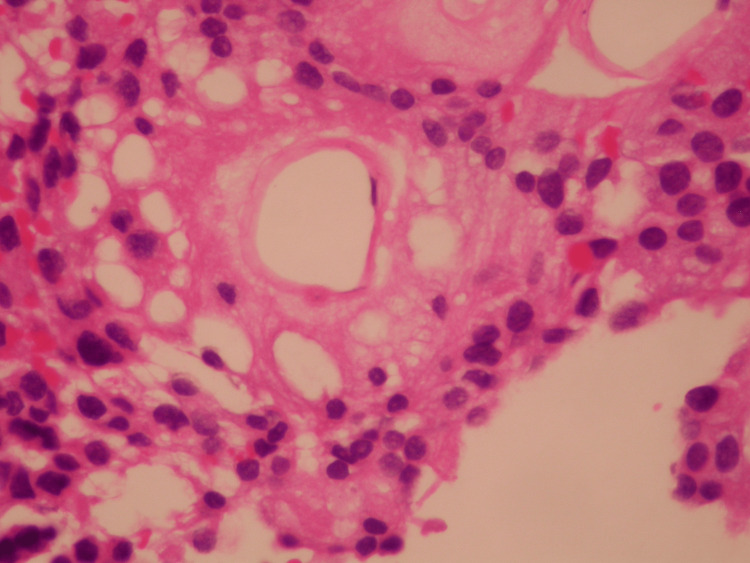
Myxopapillary ependymoma at higher magnification (×400) showing mucoid material between blood vessels and tumor cells.

**Figure 5:**
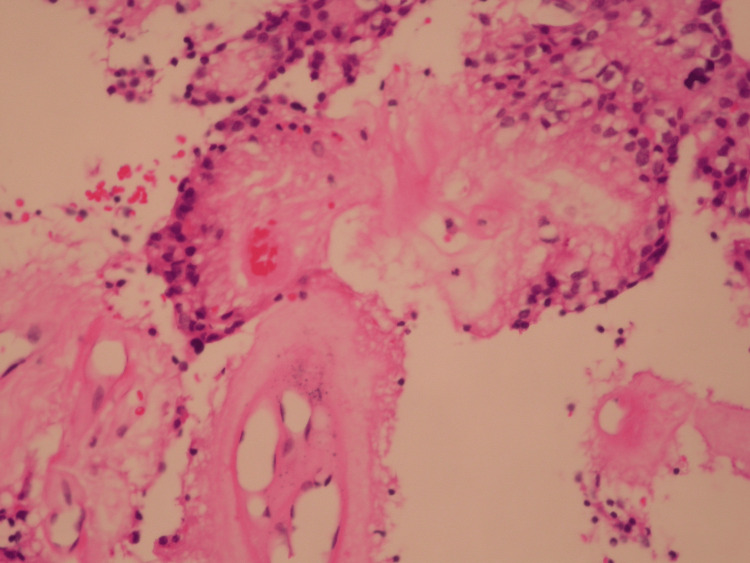
Myxopapillary ependymoma showing papillary architecture with hyalinized vascular cores surrounded by tumor cells (×200).

A fresh specimen was sent to pathology for tissue diagnosis, which revealed MPE, CNS Grade 2 (Figures 4, 5).

Post-discharge 

**Figure 6:**
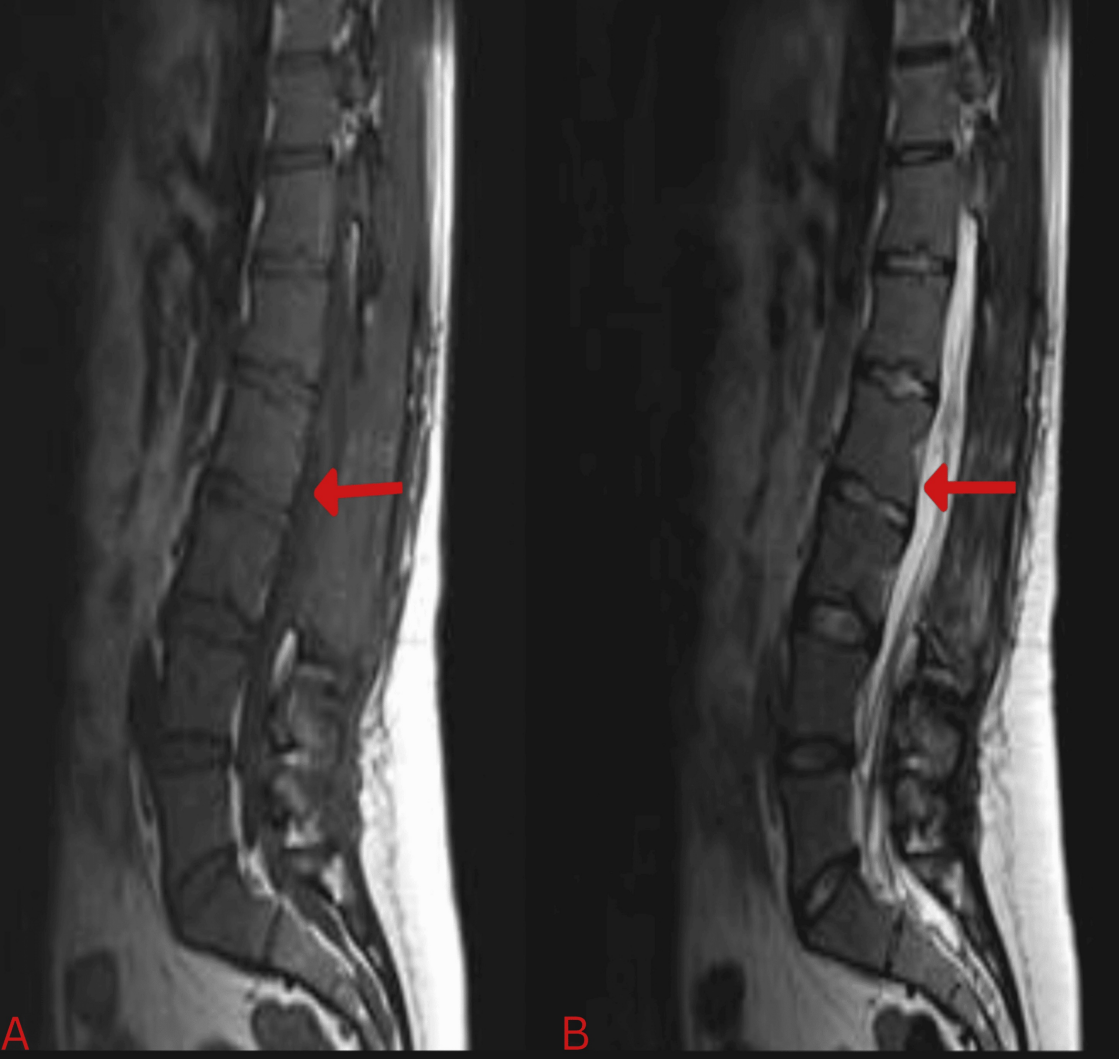
Postoperative sagittal T1-weighted (A) and T2-weighted (B) contrast-enhanced MRI of the lumbosacral spine showing complete removal of the lesion. MRI, magnetic resonance imaging

Postoperative lumbar spine MRI at three months shows post-surgical changes with no residual tumor (Figure 6). The patient returned to work and is being followed up with MRI every six months.

## Discussion

MPE is a benign and slow-growing tumor that arises from the ependymal glia of the filum terminale. It has been histologically classified as Grade 2, according to the WHO classification [3]. 

MPE is more commonly seen in adults [6], with 66.2% of tumors occurring in the lumbosacral segment and filum terminale, 31.7% in the thoracolumbar segment, and 2.1% in the cervicothoracic segment [7]. They account for 1%-5% of all spinal tumors and typically present between the ages of 30 and 50 years [7]. 

The clinical presentation of MPE is determined by the size, site, and extent of the tumor. Common symptoms include low back pain, weakness and numbness in the lower extremities, sphincteric and sexual dysfunction, and gait abnormality [3,8]. Cases of atypical presentation of MPE have been observed and could present as headaches, hydrocephalus, papilledema, visual field defects, and cranial nerve palsies [9,10,11,12]. IIP is associated with MPE and can present with headaches and papilledema [9,10]. 

Our index patient presented with features of IIP, including headaches, nausea, neck stiffness, and double vision. She also had cranial nerve palsies and lumbar CSF opening pressure >20 cmH2O. Our case was unique in that the patient presented with features of IIP without any lower limb neurology as compared to other atypical presentations observed in the literature [13].

MPEs are known to produce mucins [3], and this leads to increased CSF protein [10]. These proteins can clog up arachnoid granulations, reduce CSF absorption, and cause IIP [14]. 

Ependymomas are usually soft, encapsulated masses with an increased risk of bleeding and, therefore, can cause recurrent subarachnoid bleeding. This leads to hemosiderin deposition in the subpial layers of the brain and spinal cord and is seen as superficial siderosis on MRI (Figure 2) [15]. This explains the superficial siderosis noted in our patient’s brain MRI. 

Chronic subarachnoid bleeding may also contribute to raised intracranial pressure (ICP) by irritating or obstructing the arachnoid villi [16]. 

When MPE is suspected, spinal MRI is the diagnostic modality of choice. It appears as “a well-defined lobulated T1W iso to hypointense and T2W / STIR hyperintense solid lesion within the spinal canal with post-contrast enhancement” [3]. This was consistent with the spine MRI findings in our patient. 

Surgical excision is the gold standard treatment for spinal cord ependymomas, especially MPEs [5]. This leads to symptom resolution due to relief of neural compression and restoration of normal CSF flow dynamics. Our index patient observed this, as there was postoperative symptom relief, and they returned to work within a few weeks. 

MPEs have a high local recurrence rate of about 10%-19%, with lower recurrence rates noted in cases managed with gross total resection [5]. This highlights the need for radical initial resection and postoperative surveillance.

## Conclusions

Spinal cord MPE is a rare but important cause of increased ICP. The exact cause is yet to be proven; however, available literature suggests that tumor protein secretion and recurrent bleeding from the tumor could be important factors in the pathogenesis of this rare presentation of spinal cord MPE. This highlights the significance of spinal imaging in the workup of raised ICP if there is no immediate identifiable etiology. 
